# Right ventricular morphology and function in patients with Noonan’s syndrome after pulmonary intervention

**DOI:** 10.1186/1532-429X-13-S1-P189

**Published:** 2011-02-02

**Authors:** Jabbour Andrew, Sonia Bartolome, Albert Teis, Tevfik Ismail, Sanjay Prasad, Sylvia SM Chen, Anna Seale, Philip Kilner

**Affiliations:** 1 Royal Brompton Hospital and Imperial College, London, UK; 2 Royal Brompton Hospital, London, UK

## Introduction

The prevalence of cardiac abnormalities in Noonan’s syndrome is high. Pulmonary valve and artery stenoses commonly require intervention in this group with short and long term sequelae. Cardiovascular magnetic resonance (CMR) is the imaging modality of choice for assessment of right ventricular size and function.

## Hypothesis

We aimed to determine the prevalence and severity of right ventricular and pulmonary abnormalities by CMR in patients with Noonan’s syndrome that had undergone prior pulmonary valve or artery intervention for stenosis.

### Methods

CMR scans of patients with Noonan’s syndrome referred for imaging were analyzed. All patients were imaged on a 1.5T MRI. RV volumes were generated from SSFP cines and normalized to gender, body surface area and age. Phase contrast velocity mapping was used to assess pulmonary and aortic flow. The prevalence of concomitant cardiovascular abnormalities was also assessed.

## Results

Thirty two consecutive patients (age 29 (20 - 50), (median (IQR); male 69%) were studied. Prior intervention for MPA or PV stenosis was performed in 15 (47%) patients and data was compared to 17 (53%) Noonan’s patients without MPA or PV intervention. Pulmonary regurgitation was more prevalent (73% vs. 33%, χ^2^ = 8.4; p = 0.037) and severe (regurgitant fraction 26.4±5% (mean ± SEM) vs. 6.5 ± 3%; p<0.01) in the prior intervention group. Although indexed RV end-diastolic volumes were also greater (128 ±16 mL/m^2^ vs. 91 ± 10 mL/m^2^; p=0.04), no difference was observed in RVESV, RVEF or wall thickness, nor right atrial area (all, p=0.3). Mild to moderate PV stenosis was seen in 5 (33%) with prior intervention and 4 (24%) without. Other abnormalities included tricuspid dysplasia (n=1, 3%), anomalous pulmonary venous drainage (n=2, 6%), aortic coarctation (n=1, 3%), aortic root dilatation (n=1, 3%), myxomatous mitral valve (n=2, 6%), evidence of previous ASD repair (n=8, 25%), VSD repair (n=3, 9%) and AVSD repair (n=1,3%).

## Conclusions

CMR-determined moderate and severe pulmonary regurgitation is frequent in patients with Noonan’s syndrome after pulmonary intervention. RV function remains well preserved despite increased volume load. CMR, an ideal imaging modality for the assessment of pulmonary valve and RV function also allows detection of associated cardiac abnormalities in Noonan’s syndrome.

